# What I've Learned from E-Patients

**DOI:** 10.1371/journal.pmed.0020206

**Published:** 2005-08-09

**Authors:** Dan Hoch, Tom Ferguson

## Abstract

Hoch and Ferguson discuss what they've learned from working with an online support group for people with epilepsy.

As a neurologist subspecializing in epilepsy at a respected academic institution, I (DH) assumed that I knew everything I needed to know about epilepsy and patients with epilepsy. I was wrong.

In September of 1994, John Lester, my colleague in the Department of Neurology at Massachusetts General Hospital, showed me an online bulletin board for neurology patients that he had created [1]. In reading through the online messages, I observed hundreds of patients with neurological diseases sharing their experiences and discussing their problems with one another.

I knew that many patients with chronic diseases had been making use of online medical information [2]. Nonetheless, I was shocked, fascinated, and more than a bit confused by what I saw. I'd been trained in the old medical school style: my instructors had insisted that patients could not be trusted to understand or manage complex medical matters. Thinking back through my years of training and practice, I realized that there had always been an unspoken prohibition against groups of patients getting together. I had the uncomfortable sense that by promoting interactions between patients and de-emphasizing the central role of the physician, I might be violating some deep taboo.

## Remarkably Complex Stories

My initial doubts notwithstanding, I found dozens of well-informed, medically competent patients sharing information on a variety of topics. I was especially struck by the many stories recounting the development of a particular patient's illness, the patient's efforts to manage it, and the resulting interactions with health professionals. By telling their stories in such elaborate detail, experienced group members could offer a great deal of useful advice and guidance to those newly diagnosed, based on what they had learned in their own online research, what they had been told by their clinicians, and what they had deduced from personal experiences with the disease.

These “patient stories” often included a number of empowering elements that set them apart from the advice patients typically receive from their clinicians: role modeling by an active, critical, well-informed “expert patient” ([1]; http://patientweb.net), comparative reviews and recommendations of clinicians and treatment facilities [2–5], and advice about how to handle the practical details of living with a chronic illness [6] (such as how to organize a home medical record, manage treatment side effects, find the best drug prices, and deal with less-than-perfect health professionals and health-care provider systems, and a wide variety of other topics relating to effective medical self-management). These extended patient narratives—no two alike—thus gave rise to an accumulated body of what my colleagues and I began to think of as an expert patient knowledge base. We concluded that these patient narratives could be invaluable resources for clinicians and researchers, interested in taking an in-depth look at the changing roles of patients and clinicians in the Internet age.

The constant outpouring of sympathy and support that we observed in interactions among community members surpassed anything a patient might conceivably expect to receive at a doctor's office. As Richard Rockefeller, President of the Health Commons Institute, has suggested, disease-specific online patient networks provide their members with an invaluable type of around-the-clock support that he has called the “chicken soup of the Internet” [7].

Working with several colleagues, I initiated an observational study to analyze the ways in which E-patients were using this new medium. Since I am an epilepsy specialist, we decided to focus on an epilepsy support group at the site Lester had created, BrainTalk Communities (http://www.braintalk.org) (Figure 1) [8]. The BrainTalk Communities currently host more than 300 free online groups for neurological conditions (such as Alzheimer disease, multiple sclerosis, Parkinson disease, chronic pain, epilepsy, and Huntington disease) for patients across the globe. More than 200,000 individuals visit the BrainTalk Communities' Web site on a regular basis. This site is now owned and operated by an independent nonprofit group, BrainTalk Communities, and is no longer formally associated with Massachusetts General Hospital.

**Figure 1:**
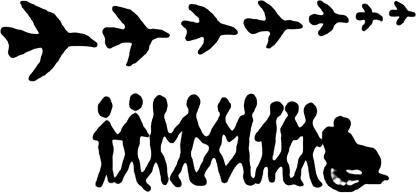
Logo of the BrainTalk Communities—Online Patient Support Groups for Neurology

What we found surprised us. We assumed that most interactions would be support related, with some members describing their medical experiences and others offering active listening, sympathy, and understanding. But while such interactions were an important part of the group process, they were observed in only about 30% of the postings. In the remaining 70% of the postings, group members provided each other with what amounted to a crash course in their shared disease, discussing topics such as the anatomy, physiology, and natural history of the disorder; treatment options and management guidelines for each form of treatment; and treatment side effects, medical self-management, the day-to-day practicalities of living with the disease, and the effects of their condition on family and friends (Table 1).

**Table 1:**
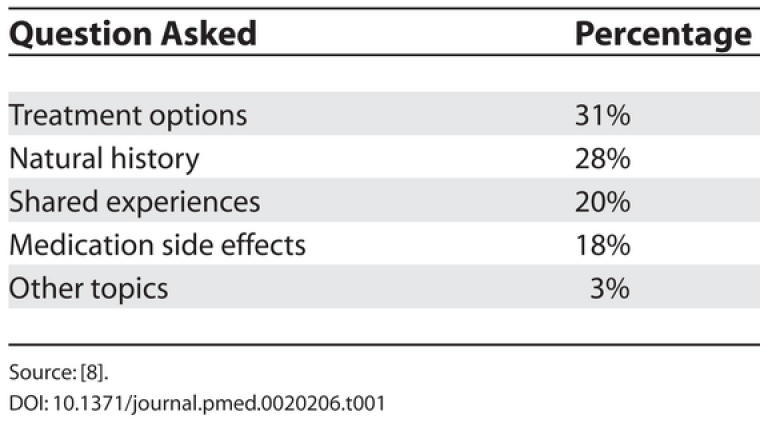
BrainTalk Communities Online Epilepsy Support Group: Types of Questions Asked by Users

Source: [8].

## A Source of Information

Much of the information that the group provided to members was similar to what I routinely provided to my own clinic patients. So I was surprised to learn that many of the clinicians caring for group members provided considerably less information, guidance, and support. And some, apparently, provided none at all. Statements such as “My provider is too busy,” “My provider doesn't care,” or “My provider doesn't seem to know about such-and-such” were alarmingly common. About 10% of the members' posts spontaneously mentioned that they had been unable to get the medical information that they needed from their own clinicians. When we surveyed members directly, more than 30% said that they had been unable to obtain all the medical information they would have liked from their physicians (Table 2). This was a primary reason for many members' participation in the group.

**Table 2:**
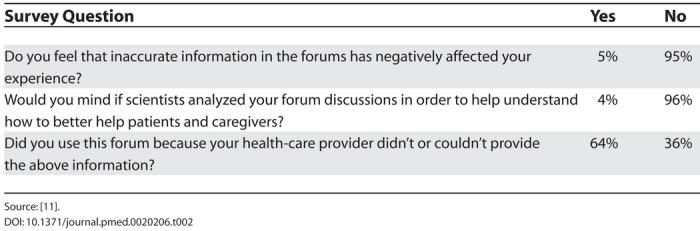
BrainTalk Communities Epilepsy Support Group: Responses to a Survey of Users

Source: [11].

Some other types of information, especially practical tips for living with epilepsy and the social aspects of the disease, went far beyond what I had been providing for my own patients. I am a board-certified epilepsy specialist at one of the most highly respected medical centers in the United States, yet I learned a great deal about these topics from the support group. I now share many of the things I learned from group members with my clinic patients.

The BrainTalk Communities epilepsy support group that we observed was facilitated by volunteer patient moderators, with little or no professional input. About 6% of the postings contained information that some of our medical reviewers considered at least partly mistaken, misinterpreted, outdated, or incomplete. We observed that other group members frequently corrected such misinformation. And group participants appeared to understand that they should not take uncorroborated statements as hard facts. They seemed well aware that some postings were erroneous, and in fact seemed to substantially overestimate the incidence of questionable materials.

We observed no serious problems as a result of these questionable postings, and saw many reports by patients who had obtained better care, prevented medical mistakes, or averted serious injury because of the information and advice they received from fellow group members. We concluded that, as Ferguson and Frydman have suggested, many professionals have seriously overestimated the risks and underestimated the benefits of online support groups and other online health resources for patients, probably because they do not operate within our familiar professionally centered constructs [9].

## What I've Learned

In retrospect, the most important thing I (DH) have learned from our online group was that patients want to know about, and in most cases are perfectly capable of understanding and dealing with, everything their physician knows about their disease and its treatments. After observing the group, I realized that I had been providing my patients with a very limited subset of what I knew about their condition. Today, there is nothing that I know about epilepsy that I would hesitate to share with a patient. For example, I now offer my patients an open and frank discussion of the very rare sudden unexpected death in epilepsy syndrome. I had previously not mentioned this rare but alarming complication, fearing that some patients might become overly concerned with it. But once I discovered that BrainTalk Communities group members discussed this topic quite openly and freely online, reviewing the scientific data in a sophisticated way, I began to share my knowledge on this topic with my clinic patients. My newfound frankness has been much appreciated. And none of my patients have become unduly troubled by these discussions.

I have also learned that an online group like the BrainTalk Communities epilepsy group is not only much smarter than any single patient, but is also smarter, or at least more comprehensive, than many physicians—even many medical specialists. While some postings do contain erroneous material, online groups of patients who share an illness engage in a continuous process of self-correction, challenging questionable statements and addressing misperceptions as they occur. And while no single resource, including physicians, should be considered the last word in medical knowledge, the consensus opinion arrived at by patient groups is usually quite excellent. And if more expert clinicians offered to consult informally with the online support groups devoted to their medical specialties—as I now do—we could help group members make information and opinion shared in these groups even better.

I had been taught to believe that patients could only be “empowered” by their clinicians. And while I do believe that clinicians can help in this regard by sharing their knowledge openly and by encouraging patient self-reliance, it now seems quite clear that growing numbers of patients are perfectly capable of empowering themselves, with or without their clinician's blessing. Physicians and other health professionals should do all they can to support them in this worthy effort.

As a result of what we've learned from these online patient networks, our research group has developed a password-protected Web site, PatientWeb (https://fisher.mgh.harvard.edu/), for the patients that we see in the clinic—all those patients with epilepsy who receive medical care at the Massachusetts General Hospital and Brigham and Women's Hospital. Thanks to what we have learned from these online groups, we plan to pilot new ways for private, local online groups made up of patients with the same disease and receiving care from the same clinicians to collaborate with each other, and with their clinicians, more effectively.

## Conclusions

Clinicians have overestimated the downsides, while seriously underestimating the benefits, of condition-specific online patient support communities. These free online resources now provide invaluable services 24 hours a day, seven days a week, for patients across the country and around the world. It would be unfortunate indeed if medical professionals let their uneasiness at this emerging trend toward patient empowerment and autonomy cloud their ability to assess the impressive benefits these groups provide.

Many patients are now ready, willing, and able to take a more active role in their own care, and the care of others with related diseases. By encouraging patients to do more for themselves and for each other, clinicians can help mitigate many of the negative effects of contemporary time-pressured medical practice. Thus, even though there may now be less time for the counseling, storytelling, support, information sharing, and empowerment-based training that was once a routine part of the typical office visit, we can now help our patients obtain such services by referring them to online patient networks.

The distributed expertise of online support groups is by no means limited to the emotional aspects of the illness and to the practical logistics of living with the disorder. It can also include current reviews of the literature, reports from the latest medical meetings, accounts of behind-the-scenes activities at the best treatment centers, sophisticated guidance on dealing with medical professionals, and excellent advice on dealing with complex aspects of medical management.

Finally, I have concluded that few, if any, physicians could have created a system like BrainTalk Communities. As a tech-savvy non-physician intimately familiar with both the inner workings of medical care and the power of information technology systems to create effective online communities, John Lester was less proprietary than most physicians are about medicine's proper professional “turf.” He was also less inhibited by professional biases regarding the potential value of the medical contributions that “unqualified” individuals might make. This is not an isolated occurrence. We suspect that the intensely professionally centered enculturation most physicians receive in their training and practice environments may render them, in the words of John Seely Brown and Paul Draguld, “blinkered if not blind” to the emergence of many promising new technocultural changes, which currently present new opportunities for health-care innovation [10]. Thus, physicians who seek to innovate in these areas might benefit greatly—as I have—from joining forces with Web developers, Net-savvy social scientists, experienced E-patients, and other colleagues unencumbered by the limiting belief systems that may result from our traditional medical training.

In light of their empowering social dynamics and volunteer economics, we suspect that patient-led online groups may prove to be a considerably more promising and sustainable health-care resource than professionally moderated therapy groups. And we are convinced that networked work teams linking patients, caregivers, and medical professionals will be an important model for future health-care innovation.
